# REG4 Independently Predicts Better Prognosis in Non-Mucinous Colorectal Cancer

**DOI:** 10.1371/journal.pone.0109600

**Published:** 2014-10-08

**Authors:** Tuomas Kaprio, Jaana Hagström, Harri Mustonen, Selja Koskensalo, Leif C. Andersson, Caj Haglund

**Affiliations:** 1 Department of Surgery, Helsinki University Central Hospital, Helsinki, Finland; 2 Research Programs Unit, Translational Cancer Biology, University of Helsinki, Helsinki, Finland; 3 Department of Pathology, Haartman Institute, University of Helsinki and HUSLAB, Helsinki, Finland; Queen Mary Hospital, Hong Kong

## Abstract

**Introduction:**

Colorectal cancer (CRC) is one of the world’s three most common cancers and its incidence is rising. To identify patients who benefit from adjuvant therapy requires novel biomarkers. The regenerating islet-derived gene (REG) 4 belongs to a group of small secretory proteins involved in cell proliferation and regeneration. Its up-regulated expression occurs in inflammatory bowel diseases also in gastrointestinal cancers. Reports on the association of REG4 expression with CRC prognosis have been mixed. Our aim was to investigate tumor REG4 expression in CRC patients and its coexpression with other intestinal markers.

**Methods:**

Tumor expression of REG4 was evaluated by immunohistochemistry in 840 consecutive surgically treated CRC patients at Helsinki University Central Hospital. Expression of MUC1, MUC2, MUC5AC, synapthophysin, and chromogranin was evaluated in a subgroup of 220 consecutively operated CRC patients. REG4 expression with clinicopathological parameters, other intestinal markers, and the impact of REG4 expression on survival were assessed.

**Results:**

REG4 expression associated with favorable clinicopathological parameters and with higher overall survival from non-mucinous CRC (p = 0.019). For such patients under 65, its expression was an independent marker of lower risk of death within 5 years that cancer; univariable hazard ratio (HR) = 0.57; 95% confidence interval (CI) (0.34–0.94); multivariable HR = 0.55; 95% CI (0.33–0.92). In non-mucinous CRC, REG4 associated with positive MUC2, MUC4, and MUC5AC expression.

**Conclusion:**

We show, to our knowledge for the first time, that REG4 IHC expression to be an independent marker of favorable prognosis in non-mucinous CRC. Our results contradict those from studies based on quantification of REG4 mRNA levels, a discrepancy warranting further studies.

## Introduction

The world’s third most common cancer, with an annual incidence of over one million new cases, is colorectal cancer (CRC) [1]. Its cure is based on early diagnosis, radical surgery, and possible adjuvant therapy. Such therapy is routine for stage III patients. At stage II, the advantage of adjuvant therapy is, however unclear, since about 80% of surgically treated stage II patients survive without chemotherapy. Although we know many high risk factors; T4-stage, low differentiation, vascular invasion, tumor obstruction, bowel perforation, and inadequate lymph node resection, we cannot always identify patients who will benefit from adjuvant therapy. It would be beneficial to find new biomarkers to aid in treatment decisions.

Regenerating islet-derived gene (REG) proteins represent a group of small secretory proteins involved in cell proliferation and regeneration, that also participate in formation of the immune system [2], [3]. They belong to the calcium-dependent lectin (C-lectin) superfamily and are divided into four families, REG I to IV, based on their primary structure. At variance with the other REG proteins, REG4 binds polysaccharides independently of calcium [4]. The genes encoding REG I to III genes are located on chromosome 2p12, while that of REG4 is on 1p12–13. REG4 was first cloned and identified by Hartupee et al [5] and by Kämäräinen et al [6]. Containing 158 aminoacids and with a molecular of weight of 18 kDA, it is physiologically expressed in the colon and the small intestine, with high expression in enteroendocrine cells. [6], [7]. In the gastrointestinal epithelium, REG4 is activated during specific phases of differentiation and maturation, its expression is spatially specific, and it has been suggested to support mucinous and neuroendocrine differentiation or [8], [9]. Up-regulated REG4 expression occurs in inflammatory bowel diseases (IBD) [6] and also occurs in many malignancies: colorectal, gastric, and pancreatic cancers [10]–[12]. REG4 is suggested to participate in carcinogenesis and tissue regeneration, to act as an antiapoptotic factor, and to promote proliferation and invasion [13]. The ultimate physiological and pathological roles of REG4 still remain elusive, however. Expression of REG4 in GI-tract cancers has in several studies appeared to be of predictive and prognostic value [10], [11], [14], [15]. The findings in CRC have, though, been controversial: increased expression is a sign of poor prognosis according to Numata et al [16], but no association with prognosis has emerged in other studies [17], [18].

This study aimed to evaluate in CRC the role of REG4 as a prognostic marker and its association with clinicopathological parameters in a cohort of 840 patients. Furthermore, in a subgroup of 220 patients, we focused on association of REG4 expression with other markers: markers of mucinous differentiation MUC1, MUC2, MUC4, and MUC5AC, and also markers of neuroendocrine phenotype: synaptophysin and chromogranin.

## Methods

### Patients

The study population comprised 840 consecutive colorectal cancer patients undergoing surgery in 1983–2001 at the Department of Surgery, Helsinki University Central Hospital. A subgroup of 240 comprised consecutively operated patients between 1998–2001. REG4 was studied in the whole patient series, and the other markers studied in the subgroup. The Finnish Population Register Centre provided the follow-up vital-status data needed to compute survival statistics, and Statistics Finland provided cause of death for all those deceased. Median age at diagnosis was 66, with a median follow-up of 5.1 years (range 0–25.8). The 5-year disease-specific survival rate was 58.9% (95% Cl 55.0–62.8%). For the subgroup, median age at diagnosis was 67 with a median follow-up of 6.0 years (range 0–13.2). The 5-year disease-specific survival rate was 64.8% (95% Cl 58.1–71.5%). Clinicopathological characteristics of both groups are in S1.

This study complies with the Declaration of Helsinki and was approved by the Surgical Ethics Committee of Helsinki University Central Hospital (Dnro HUS 226/E6/06, extension TMK02 §66 17.4.2013) and the National Supervisory Authority of Welfare and Health gave the permission to use tissue samples without individual informed consent in this retrospective study (Valvira Dnro 10041/06.01.03.01/2012).

### Tissue microarray

Formalin-fixed and paraffin-embedded tumor samples came from the archives of the Department of Pathology, Helsinki University Central Hospital. Representative areas of tumor samples on hematoxylin- and eosin-stained slides were marked by an experienced pathologist. Three 1.0-mm-diameter punches taken from each sample were mounted on each recipient paraffin block with a semiautomatic tissue microarray instrument (TMA) (Beecher Instruments, Silver Spring, MD) as described [19].

### Immunohistochemistry

TMA- and tissue-blocks were freshly cut into 4-µm sections. After deparaffinization in xylene and rehydration through a gradually decreasing concentration of ethanol to distilled water, the slides were treated in a PreTreatment module (Lab Vision Corp., Fremont, CA) in pre-treatment buffer for 20 minutes at 98°C for antigen retrieval. The staining procedure by the Dako REAL EnVision Detection system, Peroxidase/DAB+, Rabbit/Mouse (Dako, Glostrup, Denmark) used an Autostainer 480 (Lab Vision). Tissues were incubated with primary antibodies for one hour at room temperature. The REG4 antibody is as described in [20], others were from commercial sources. Antibodies and their dilutions are in Table 1.

**Table 1:** Antibodies for immunohistochemistry.

Antibody	Clone	Company	Pre-treatment	Dilution	Positive control
**REG4**	mAb	In-house	Tris-HCl (pH 8.5)	1∶50	Colon
**MUC1**	mAb, Ma552	Novocastra, UK	Citrate (pH 6.0)	1∶25	Stomach
**MUC2**	mAb, Ccp58	Novocastra, UK	Citrate (pH 6.0)	1∶100	Colon
**MUC4**	mAb, 1G8	Invitrogen,USA	Tris-EDTA (pH 9.0)	1∶100	Colon
**MU5AC**	mAb, CLH2	Novocastra, UK	Citrate (pH 6)	1∶50	Stomach
**Synaptophysin**	mAb 27G12	Novocastra, UK	Tris-EDTA (pH 9.0)	1∶200	Colon
**Chromogranin**	mAb, 5H7	Novocastra, UK	Tris-EDTA (pH 9.0)	1∶2000	Colon

REG4 antibody described in detail in reference [20] mAb = monoclonal antibody. Antibody host: mouse.

### Scoring of samples

**Figure 1:**
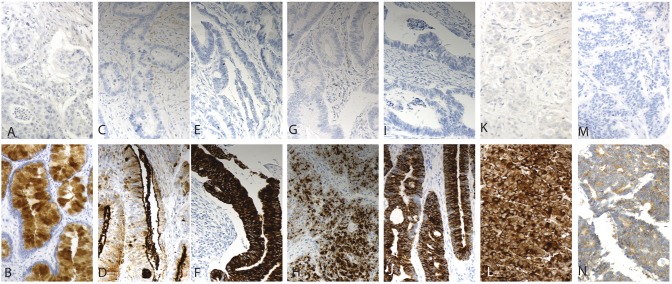
Immunohistochemical staining patterns of the antibiodies studied. Representative images of antibody stainings in colorectal cancer; REG4-negative and -positive (A & B), MUC1- negative and -positive (C & D); MUC2-negative and -positive (E & F), MUC4-negative and -positive (G & H), MUC5AC-negative and -positive (I & J), synapthophysin-negative and -positive (K & L), chromogranin-negative and -positive (M & N) Original magnification was x 20.

REG4 cytoplasmic expression was scored in tumor cells as either negative of positive. MUC1 and MUC2 expressions were cytoplasmic in tumor cells and were scored as negative-low-moderate-high according to intensity. For further statistical analysis they were grouped into: low (negative to low) and high (moderate to high). MUC4 and MUC5AC cytoplasmic expressions were scored either negative or positive. Neuroendocrinic differentiation (negative vs positive) was evaluated by cytoplasmic synapthophysin and chromogranin positivity. Stainings were scored independently by T.K. and J.H., who were blinded to clinical data and outcome. Differences in scoring were discussed until consensus. Representative images of expression are in Figure 1. REG4 expression was compared with proliferation index by Ki-67 staining, which we published previously [21].

### Statistical analyses

Evaluation of the association between REG4 expression and clinicopathological parameters or MUC expressions was done by the exact Pearson chi-square test or the exact linear-by-linear association test for ordered parameters. Disease-specific overall survival was counted from date of surgery to date of death from colorectal cancer, or until end of follow-up. Survival analysis by the Kaplan-Meier method was compared by the log rank test. The Cox regression proportional hazard model served for uni- and multivariable survival analysis, adjusted for sex, age, Dukes classification, and differentiation. Testing of the Cox model assumption of constant hazard ratios over time involved the inclusion of a time-dependent covariate separately for each testable variable. The hazard ratio of differentiation was analyzed in two periods (0 to 1.25 and 1.25 to 5 years) in order to meet the assumptions of the Cox model, with the time-dependent Cox model. Interaction terms were considered and an interaction between REG4 expression and age emerged. We therefore calculated the prognostic role of REG4 separately for patients under and over 65. All tests were two-sided. A p-value of 0.05 was considered significant. All statistical analyses were done with SPSS version 20.0 (IBM SPSS Statistics, version 20.0 for Mac; SPSS, Inc., Chicago, IL).

## Results

### Immunohistochemistry

REG4 expression in tumor cells was cytoplasmic and slightly granular. When present, expression was evident in the vast majority of tumor cells, but with no nuclear expression. In whole tissue sections, no clear distinction in expression appeared between the invasive front and the rest of the tumor. Moreover, in whole sections, REG4 expression appeared in some cases in normal epithelium, but was down-regulated in tumor cells. Expression of mucins, synapthopysin, and chromogranin was cytoplasmic, with no nuclear expression.

Of the 840 tumors represented in the TMA, REG4 staining could be evaluated in 793; 580 (73.1%) were scored as negative and 213 (28.9%) scored as positive. In a subgroup of 220 tumors, MUC 1 expression was evaluated in 206 (low 83.5% and high 17.5%), MUC2 expression in 210 (low 17.1% and high 82.9%), MUC4 expression in 208 (negative 51.0% and positive 49.0%), and MUC5AC expression in 205 (negative 93.2% and positive 6.8%). Neuroendocrinic positivity (either/both synaptohysin- and chromogranin-positive) could be evaluated in 212 (negative 92.9% and positive 7.1%).

### REG4 in lymph-node metastasis

**Figure 2:**
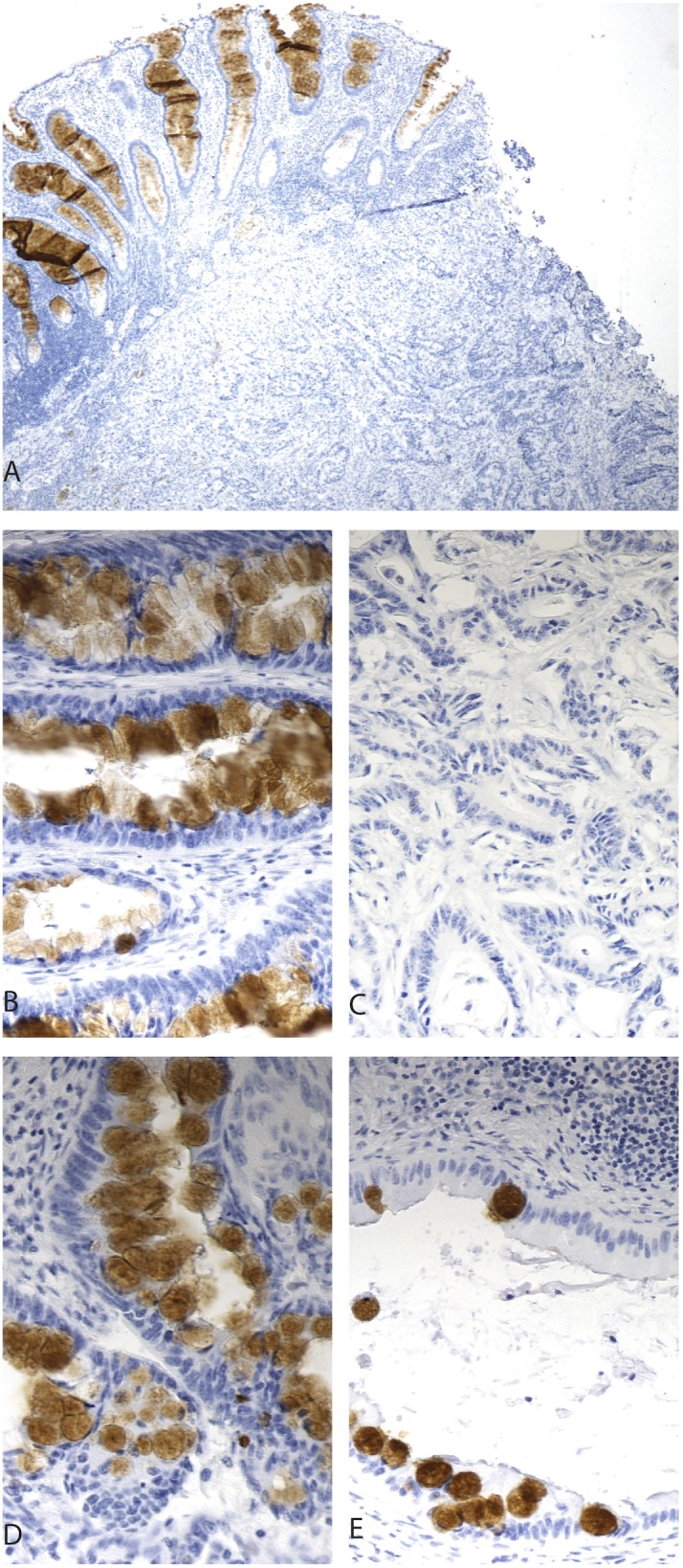
REG4 expression in primary tumors and lymph-node metastases. REG4-negative expression in tumor and -positive in adjacent epithelium (A). Positive REG4 expression in primary tumor (B) and negative in its corresponding lymph node metastasis (C).Positive REG4 expression in primary tumor (D) and positive in its corresponding lymph node metastasis (E). Original magnification x 4 in figure A and x 20 in B–E.

Based on TMA results, we chose 10 patients with Dukes C disease, 5 with REG4-positive tumors and 5 with negative tumors. None of the patients’ REG4 negative tumors showed positivity in their lymph-node metastasis, whereas of the 5 patients with REG4-positive tumor, 2 showed positivity also in their lymph node metastasis (Figure 2).

### REG4 and clinicopathological parameters

Cytoplasmic REG4 expression associated with less-advanced stage (p = 0.014), location in the right hemicolon (p = 0.035), and mucinous histology (p<0.0001). We saw no association with age, gender, differentiation, or location (colon vs. rectum) (Table 2). As REG4 associated strongly with tumor histology, we analyzed mucinous and non-mucinous CRC separately. In non-mucinous CRC, REG4 expression associated with less advanced stage (p = 0.005) and higher differentiaton (p = 0.023). No association appeared with age, gender, tumor location, nor tumor side. (Table 3) In mucinous CRC, REG4 expression did not associate with any clinocopathological parameter (data not shown). No association between REG4 and Ki-67 was seen in CRC nor in any of the subgroups analyzed (data not shown).

**Table 2:** Association between REG4 expression and clinicopathologic parameters in colorectal cancer.

REG4 expression			
	negative	positive	
n (%)	580 (73.1)	213 (28.9)	p-value
**Age. years**			
<65	245 (42.2)	94 (44.1)	0.686
≥65	335 (57.8)	119 (55.9)	
**Gender**			
Male	313 (54.0)	128 (60.1)	0.124
Female	267 (46.0)	85 (39.9)	
**Dukes**			
A	75 (12.9)	44 (20.7)	0.014
B	202 (34.8)	73 (34.3)	
C	164 (28.3)	55 (25.8)	
D	139 (24.0)	41 (19.2)	
**Grade (WHO)**			
1	14 (2.4)	13 (6.2)	0.064
2	394 (68.3)	150 (71.1)	
3	150 (26.0)	38 (18.0)	
4	19 (3.3)	10 (4.7)	
Missing			
**Location**			
Colon	294 (50.7)	116 (54.5)	0.346
Rectum	286 (49.3)	97 (45.5)	
**Side**			
Right	147 (25.3)	70 (32.9)	0.035
Left	433 (74.7)	143 (67.1)	
**Histology**			
Adenomatous	539 (92.9)	173 (81.6)	<0.0001
Mucinous	41 (7.1)	39 (18.4)	

Exact Pearson chi-square test for 2×2 tables and exact linear-by-linear association test for tables with ordered variables. Missing data excluded from analyses.

**Table 3:** Association between REG4 expression and clinicopathologic parameters in non-mucinous colorectal cancer.

REG4 expression			
	negative	positive	
n (%)	539	173	p-value
**Age. years**			
<65	228 (42.3)	76 (43.9)	0.706
≥65	311 (57.7)	97 (56.1)	
**Gender**			
Male	289 (53.6)	101 (58.4)	0.273
Female	250 (46.4)	72 (41.6)	
**Dukes**			
A	74 (13.7)	39 (22.5)	0.005
B	186 (34.5)	59 (34.1)	
C	148 (27.5)	46 (26.6)	
D	131 (24.3)	29 (16.8)	
**Grade (WHO)**			
1	13 (2.4)	11 (6.4)	0.023
2	375 (70.0)	124 (72.5)	
3	132 (24.6)	32 (18.7)	
4	16 (3.0)	4 (2.3)	
Missing			
**Location**			
Colon	268 (49.7)	87 (50.3)	0.897
Rectum	271 (50.3)	86 (49.7)	
**Side**			
Right	126 (23.4)	49 (28.3)	0.189
Left	413 (76.6)	124 (71.7)	

Exact Pearson chi-square test for 2×2 tables and exact linear-by-linear association test for tables with ordered variables. Missing data excluded from analyses.

### REG4 and other intestinal markers

In the subgroup of 220 tumors, we found that REG4 expression significantly associated with higher expression of MUC2, MUC4, and MUC5AC, but not with MUC1 expression. Nor did REG4 expression associate with markers of neuroendocrine differentiation. The same remained true when non-mucinous CRC was analyzed alone (Table 4). In mucinous CRC, REG4 expression associated with no other markers studied (data not shown).

**Table 4:** Association of REG4 expression with other biomarkers in colorectal cancer and non-mucinous colorectal cancer.

REG4 expression						
Colorectal cancer				Non-mucinous colorectal cancer		
	negative	positive		negative	positive	
n (%)	162 (76.4)	50 (23.6)	p-value	157 (78.5)	43 (21.5)	p-value
**MUC1 expression**						
low	131 (84.0)	36 (78.3)	0.368	127 (84.1)	31 (79.5)	0.492
high	25 (16.0)	10 (21.7)		24 (15.9)	8 (20.5)	
**MUC2 expression**						
low	147 (93.6)	24 (48.0)	<0.0001	143 (94.1)	22 (51.2)	<0.0001
high	10 (6.4)	26 (52.0)		9 (5.9)	21 (48.8)	
**MUC4 expression**						
neg	90 (57.0)	14 (29.2)	0.001	88 (57.5)	13 (31.7)	0.003
pos	68 (43.0)	34 (70.8)		65 (42.5)	28 (68.3)	
**MUC5AC expression**						
neg	150 (96.2)	38 (82.6)	0.004	147 (97.4)	31 (79.5)	<0.0001
pos	6 (3.8)	8 (17.4)		4 (2.6)	8 (20.5)	
**Neuroendocrine differentiation**						
Negative	152 (93.8)	45 (90.0)	0.354	147 (93.6)	38 (88.4)	0.323
Positive	10 (6.2)	5 (10.0)		10 (6.4)	5 (11.6)	

Exact pearson chi-square test for 2×2 tables. Missing data excluded from analyses.

### Survival analysis

**Figure 3:**
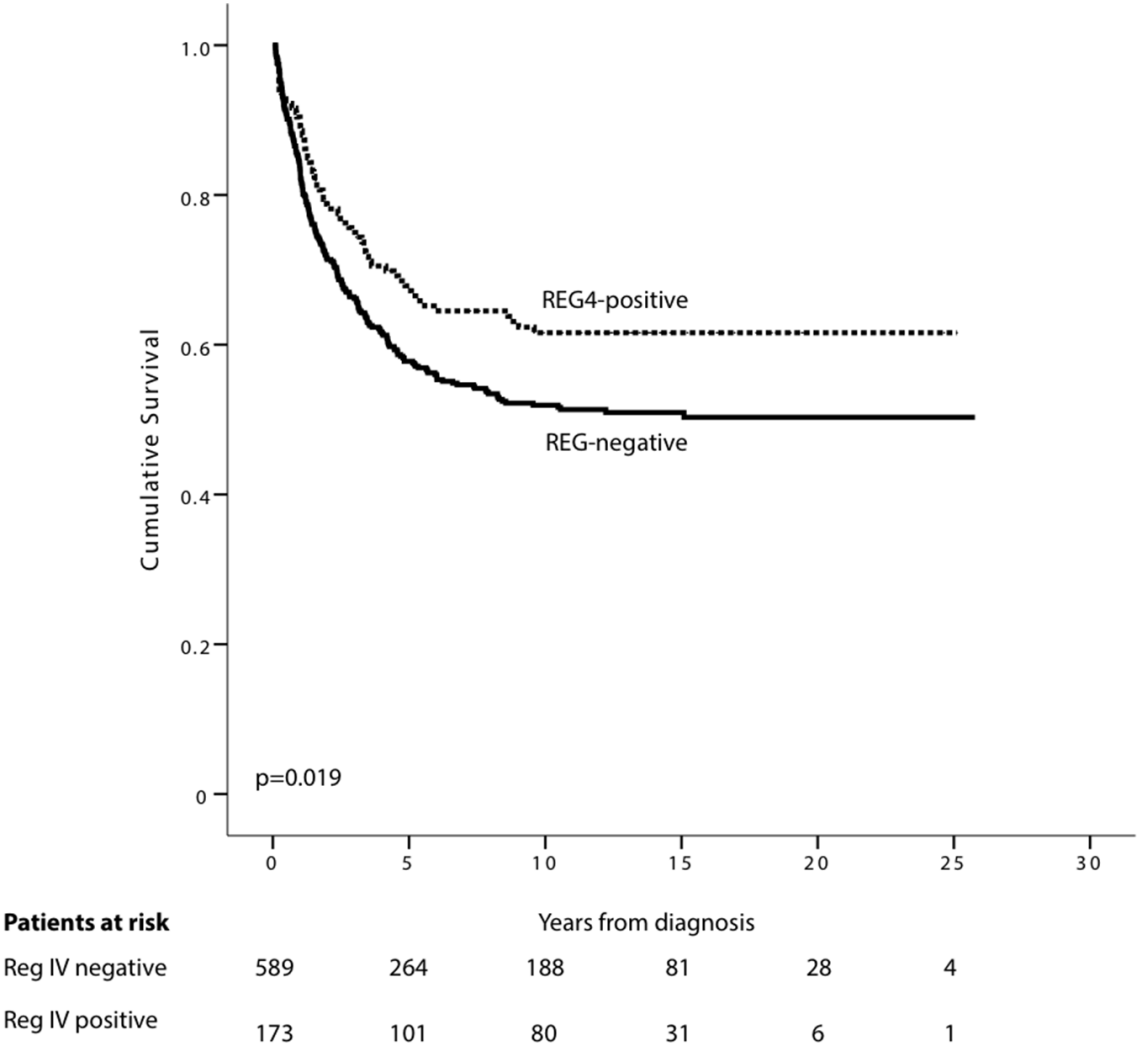
REG4 expression indicating better prognosis in non-mucinous colorectal cancer. Disease-specific survival analysis according to the Kaplan-Meier method for REG4 expression in non-mucinous colorectal cancer by the log-rank test.

In non-mucinous, CRC REG4 positivity was a sign of favorable prognosis (p = 0.019, log-rank test); 5-year DSS for patients with positive cytoplasmic REG4 tumor expression was 67.9% (95% CI 60.5–75.3) compared to 57.8% (95% CI 53.5–62.1) for those with no cytoplasmic expression (Figure 3). In mucinous CRC, no difference appeared (data not shown). When we stratified non-mucinous CRC for patients under or over 65, REG4 expression was a sign of favorable prognosis in patients under 65 (p = 0.049, log-rank test.), with no difference for those older (p = 0.195, log-rank-test). (S2 & S3).

Cox regression univariable analyses confirmed these results, with REG4 expression being a sign of a reduced risk of death within 5 years for non-mucinous CRC. A significant difference emerged also for patients under 65 (HR 0.57, 95% CI 0.34–0.94, p = 0.029) but not for those older (HR 0.83, 95% CI 0.57–1.22, p = 0.34). Cox regression multivariable analysis for non-mucinous CRC patients under 65, adjusted for gender, stage, and differentiation, showed that REG4 was an independent factor of favorable prognosis (HR 0.55, 95% CI 0.33–0.92, p = 0.022) (Table 5).

**Table 5:** Cox uni-and multivariable analysis of relative risk of death from non-mucinous colorectal cancer within 5 years by REG4 expression for patients under 65.

REG4 expression	HR (95% CI)	P-value	N(events)	HR (95% CI)	P-value	N(events)
	Univariable			Multivariable		
Negative	1.00		228 (83)	1.00		227 (83)
Positive	0.57 (0.34–0.94)	0.029	76 (18)	0.55 (0.33–0.92)	0.022	76 (18)

Abbreviations: CI = confidence interval, HR = hazard ratio. Multivariable analysis included adjustment for gender, Dukes class, differentiation grade (G1/2 vs G3/4).

## Discussion

Here we show by immunohistochemistry that REG4 expression in non-mucinous colorectal cancer associates with favorable clinicopathological parameters and that REG4 is an independent marker of favorable prognosis in patients under 65. REG4 expression associates with expression of other intestinal markers: MUC1, MUC2, and MUC5AC.

REG4 expression was higher in low-stage tumors and in those with of mucinous histology. With mucinous tumors excluded, REG4 expression associated significantly with higher differentiation and low stage. REG4 expression also associated with MUC1, MUC2, and MUC5AC, which supports the finding that REG4-positive tumors are more highly differentiated than are those that are REG4 negative. These results are in accordance with findings of Li et al [17], who showed in that for REG4, immunohistochemical (IHC) expression in CRC associates significantly with higher differentiation and with absence of venous invasion. Moreover, REG4 expression showed a trend like association with low T-stage, absence of lymph node metastasis, and local disease (Dukes A-B vs C-D). Similar results appear for gallbladder cancer, where positive REG4 IHC expression, associates with higher tumor differention [22]. Controversial results for CRC in Numata et al. show that higher REG4 mRNA expression associates with higher differentiation, deeper invasion (T-stage), lymphatic invasion, presence of liver metastasis, and more advanced stage [16]. They, however measured mRNA levels by PCR, not as we did actual protein expression.

Association between REG4 levels and carcinoma has been under study in both serum and tissues. Elevated serum concentration of REG4 in carcinoma patients compared to those in healthy controls has been a finding in pancreatic cancer [12], gastric cancer [11] and gallbladder cancer [22], indicating that serum REG4 could serve as a diagnostic biomarker. Increased REG4 IHC expression has been suggested as a marker of poor prognosis in gastric cancer [23], and elevated tissue levels of REG4 mRNA may be a marker of poor prognosis in CRC [16]. REG4 IHC expression has shown, however, no effect on prognosis in CRC [17], [18]. One reason for this might be that mucinous and non-mucinous cancers were not analyzed separately. Also differences in antibodies, staining procedures, and analysis of stainings might have differed from our study. In gallbladder cancer, REG4 IHC expression has been associated with better prognosis [22].

Our results show that REG4 IHC expression is a marker of favorable prognosis in non-mucinous CRC, whereas no difference emerged in mucinous CRC. REG4 expression is constitutively high in mucinous tumors (i.e. Pseudomyxoma peritonei, and mucinous cystadenomas and mucocellular gastric cancer) This may explain why no clear variation in REG4 expression was found by IHC in the group of mucinous CRC tumours. Differences in REG4 expression was on the other hand apparent in non-mucinous CRC. In our patients under 65, elevated REG4 was an independent factor for better prognosis in non-mucinous CRC. It thus seems plausible that REG4 mRNA levels may be elevated in CRC patients with poor prognosis, but this is not translated to protein. Further studies are thus warranted to compare REG4 mRNA levels with REG4 IHC case by case.

Our results regarding five pairs of REG4-positive primary tumors and their corresponding lymph-node metastases showed that of five lymph nodes, only two showed REG4 expression; this may imply that a REG4-negative subpopulation of tumor cells is more likely to metastasize than REG4-positive cells.

REG4, is expressed in inflammatory bowel diseases and also in the margins of peptic ulcers, is considered a marker of inflammation [6]. In some of our whole-tissue sections tumor tissue stained negative for REG4, but the adjacent benign epithelium expressed REG4 strongly, apparently representing an inflammatory reaction against the tumor.

It is noteworthy that REG4 expression confers a more favorable prognosis only in CRC without mucinous differentiation. In fact, high expression of REG4 has appeared in aggressive forms of gastrointestinal cancer that show mucinous phenotype-like mucocellular (signet ring cell) carcinoma of the stomach [9]. On the other hand, REG4 is also abundantly present in mucin-rich cystadenomas of the appendix and in its malignant, disseminated form, pseudomyxoma peritonei, which is notoriously therapy resistant.

A majority of entero-endocrine cells in normal intestinal mucosa display high physiological expression of REG4 with co-expression of synaptophysin and chromogranin. Intestinal neuro-endocrine tumors of both low and high grade also show REG4 positivity, frequently with a peculiar anatomical distribution with the strongest expression in a single layer of cells in the periphery of the tumor that are in intimate contact with the surrounding stroma [6] Given this, it was somewhat intriguing that synaptophysin- and chromogranin-positive CRC tumors in this study remained negative for REG4.

Several reports suggest the oncogenic role of REG4 in the development of cancer in the gastrointestinal tract. The ultimate molecular mechanisms have, however, remained elusive. Bishnupari et al [24] reported that treatment of cultured colon adenocarcinoma cells with recombinant REG4 protein induced phosphorylation of the EGF receptor and Akt. They suggested that REG4 is a transactivator of the EGFR/Akt signaling pathway. A further elucidation of the role of exogenous REG4 as a regulator of cell growth potential is, however, awaiting identification of the putative REG4 receptor. No evidence shows that increased expression of REG4 by itself induces cancerous growth, however. Our observations on mice transgenic for human REG4 cDNA under the villin promoter that leads to global overexpression of REG4 in the intestinal mucosa did not induce increased tumor formation or mucosal hyperplasia (unpublished data).

Regulation of REG4 expression is still poorly understood. We originally reported strongly up-regulated expression of REG4 also in inflamed IBD mucosa of IBD-like foci of gastritis-induced intestinal metaplasia in the stomach [6]. This suggests that inflammatory cytokines may influence REG4 expression. Neuroendocrine differentiation, on the other hand, as seen in enteroendocrine cells, appears to confer constitutive expression of REG4. We recently reported co-expression of REG4 with the neuronal transcription factor Hath-1 (atonal, Math-1) in neuroendocrine tumors [9]. What remains to be established is whether one regulator of REG4 expression is Hath-1.

Compared to whole tissue sections, the TMA technique allows analysis of larger patient cohorts, but with a smaller proportion of the tumors evaluated. This posed no problem here, however, as the REG4 staining pattern in our whole section staining was homogenous. For technical reasons, up to 5% of the specimens were lost in the TMA-production and -staining process. The strength of this study is a large, well-characterized CRC-patient cohort with our long follow-up time.

Here we show, to our knowledge for the first time, that REG4 IHC expression is an independent marker of favorable prognosis in non-mucinous colorectal cancer in patients under 65. These results are in disagreement with those obtained by evaluating mRNA levels; our discrepancies with others’ findings warrant further studies.

## Supporting Information

Figure S1 Clinicopathologic characteristics of the study population and subgroup population.
(DOCX)

Figure S2 REG4 expression indicating better prognosis in non-mucinous colorectal cancer for younger patients. Disease-specific survival analysis according to the Kaplan-Meier method for REG4 expression in non-mucinous colorectal cancer in patients under 65 by the log-rank test.
(TIFF)

Figure S3 REG4 expression showing no difference in survival in non-mucinous colorectal cancer for older patients. Disease-specific survival analysis according to the Kaplan-Meier method for REG4 expression in non-mucinous colorectal cancer in patients 65 and older by the log-rank test.
(TIFF)
