# Supplementary material for: REG4 Independently Predicts Better Prognosis in Non-Mucinous Colorectal Cancer
Source: PLoS One. 2014 Oct 8;9(10):e109600. doi: 10.1371/journal.pone.0109600 (PMC4190354; doi:10.1371/journal.pone.0109600)
Supplement: Figure S1 — Clinicopathologic characteristics of the study population and subgroup population. (DOCX) [file pone.0109600.s001.docx]

**S1 Clinicopathologic characteristics of the study population and subgroup population**

|  | **Study population** | **Subgroup of 1998-2001** |
| --- | --- | --- |
| **n (%)** | **840** | **220** |
| **Age. years** |  |  |
| <65 | 360 (42.9) | 101 (45.9) |
| ≥65 | 480 (57.1) | 119 (54.1) |
| **Gender** |  |  |
| Male | 466 (55.5) | 134 (60.9) |
| Female | 374 (45.5) | 86 (49.1) |
| **Dukes** |  |  |
| A | 125 (14.9) | 34 (15.5) |
| B | 294 (35.0) | 71 (32.3) |
| C | 231 (27.5) | 70 (31.8) |
| D | 190 (22.6) | 45 (20.5) |
| **Grade (WHO)** |  |  |
| 1 | 29 (3.5) | 8 (3.7) |
| 2 | 571 (68.4) | 165 (76.7) |
| 3 | 202 (24.2) | 37 (17.2) |
| 4 | 33 (4.0) | 5 (2.3) |
| Missing | 5 | 5 |
| **Location** |  |  |
| Colon | 429 (51.1) | 87 (39.5) |
| Rectum | 411 (48.9) | 133 (60.5) |
| **Side** |  |  |
| Right | 227 (27.0) | 50 (22.7) |
| Left | 613 (73.0) | 170 (77.3) |
| **Histology** |  |  |
| Adenomatous | 749 (89.3) | 208 (95.0) |
| Mucinous | 90 (10.7) | 11 (5.0) |
| Missing | 1 | 1 |
