# Supplementary figures and images for: REG4 Independently Predicts Better Prognosis in Non-Mucinous Colorectal Cancer
Source: PLoS One. 2014 Oct 8;9(10):e109600. doi: 10.1371/journal.pone.0109600 (PMC4190354; doi:10.1371/journal.pone.0109600)

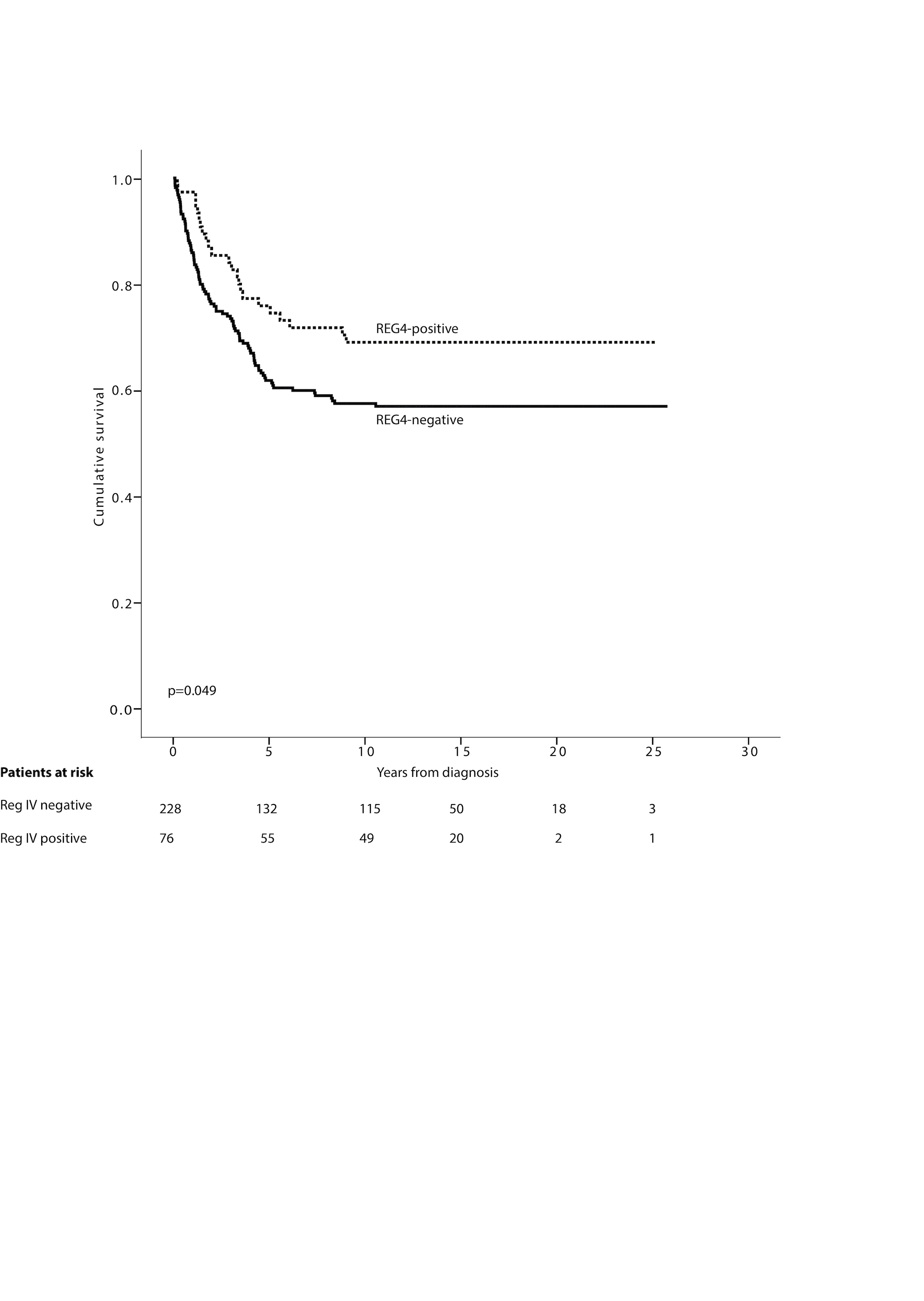

Supplement: Figure S2 — REG4 expression indicating better prognosis in non-mucinous colorectal cancer for younger patients. Disease-specific survival analysis according to the Kaplan-Meier method for REG4 expression in non-mucinous colorectal cancer in patients under 65 by the log-rank test. (TIFF) [file pone.0109600.s002.tiff]

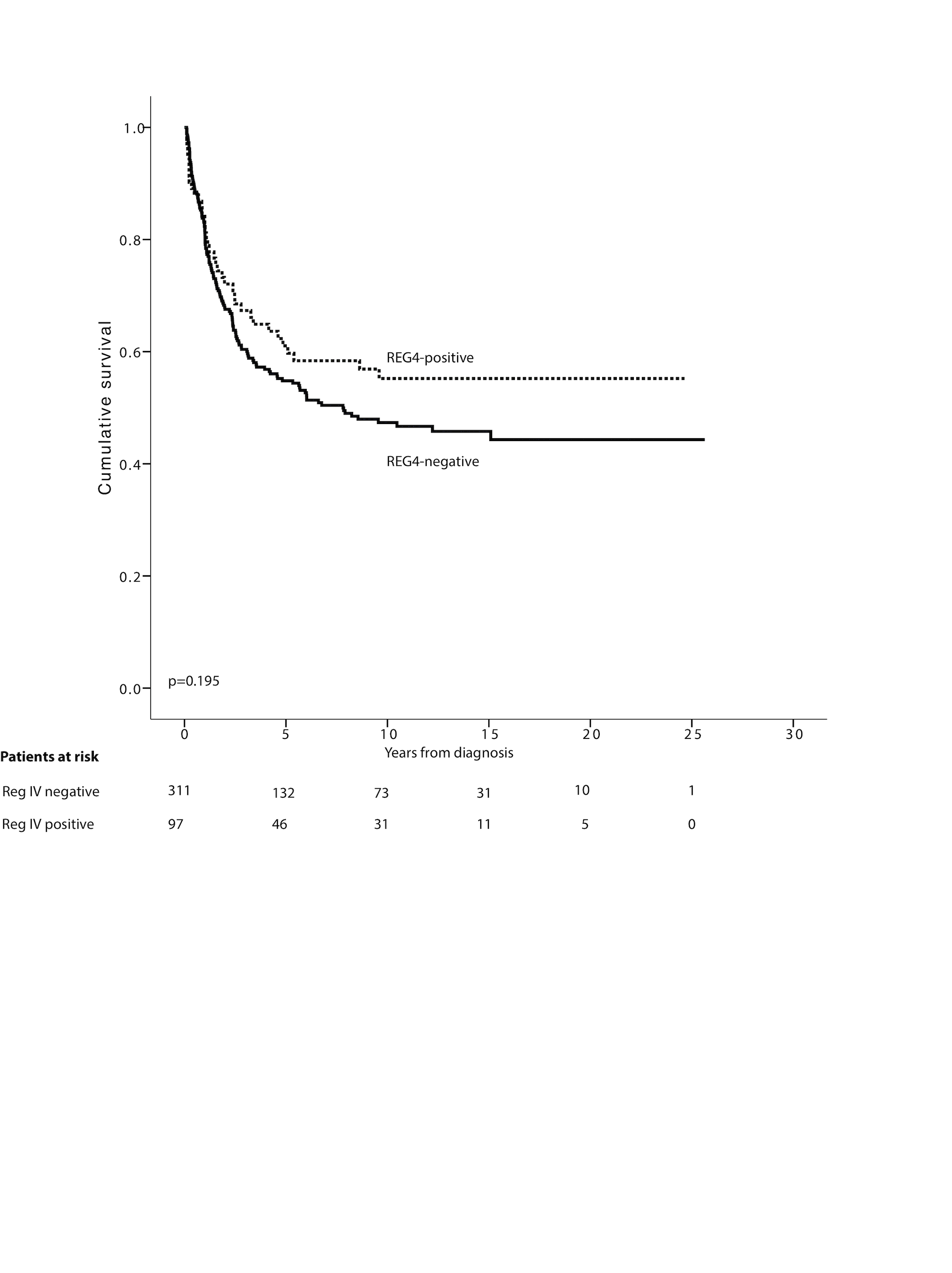

Supplement: Figure S3 — REG4 expression showing no difference in survival in non-mucinous colorectal cancer for older patients. Disease-specific survival analysis according to the Kaplan-Meier method for REG4 expression in non-mucinous colorectal cancer in patients 65 and older by the log-rank test. (TIFF) [file pone.0109600.s003.tiff]
